# Beta-Catenin Phosphorylated at Threonine 120 Antagonizes Generation of Active Beta-Catenin by Spatial Localization in trans-Golgi Network

**DOI:** 10.1371/journal.pone.0033830

**Published:** 2012-04-12

**Authors:** Cheng Du, Chuanyou Zhang, Zhuo Li, Md. Helal Uddin Biswas, K. C. Balaji

**Affiliations:** 1 Department of Surgery, University of Massachusetts Medical School, Worcester, Massachusetts, United States of America; 2 Department of Urology and Institute of Regenerative Medicine, Wake Forest University, Winston Salem, North Carolina, United States of America; The University of Kansas Medical Center, United States of America

## Abstract

The stability and subcellular localization of beta-catenin, a protein that plays a major role in cell adhesion and proliferation, is tightly regulated by multiple signaling pathways. While aberrant activation of beta-catenin signaling has been implicated in cancers, the biochemical identity of transcriptionally active beta-catenin (ABC), commonly known as unphosphorylated serine 37 (S37) and threonine 41 (T41) β-catenin, remains elusive. Our current study demonstrates that ABC transcriptional activity is influenced by phosphorylation of T120 by Protein Kinase D1 (PKD1). Whereas the nuclear β-catenin from PKD1-low prostate cancer cell line C4-2 is unphosphorylated S37/T41/T120 with high transcription activity, the nuclear β-catenin from PKD1-overexpressing C4-2 cells is highly phosphorylated at T120, S37 and T41 with low transcription activity, implying that accumulation of nuclear β-catenin alone cannot be simply used as a read-out for Wnt activation. In human normal prostate tissue, the phosphorylated T120 β-catenin is mainly localized to the trans-Golgi network (TGN, 22/30, 73%), and this pattern is significantly altered in prostate cancer (14/197, 7.1%), which is consistent with known down regulation of PKD1 in prostate cancer. These *in vitro* and *in vivo* data unveil a previously unrecognized post-translational modification of ABC through T120 phosphorylation by PKD1, which alters subcellular localization and transcriptional activity of β-catenin. Our results support the view that β-catenin signaling activity is regulated by spatial compartmentation and post-translational modifications and protein level of β-catenin alone is insufficient to count signaling activity.

## Introduction

Beta-catenin is a dual functional molecule which plays a structural role in cell-cell adhesion and a mediator for cellular signaling pathways [1]. β-catenin bridges the epithelial cell surface to cytoskeletal networks by binding to membranous molecule E-cadherin and a-catenin, which links to F-actin and cytoskeleton [1]. The catenin/cadherin protein complex is necessary to establish and maintain cell-cell adhesion [2]. On the other hand, β-catenin is also a co-activator in transcription activities through Wingless/Wnt and androgen receptor signaling pathways in prostate epithelial cells by forming a complex with either T-cell factor (TCF) or androgen receptor [1], [3], [4]. β-catenin has 12 Armadillo repeats in the middle of its sequence which form a superhelix of helices that create a positively charged groove to interact with many of its negatively charged ligands [5], [6]. This versatility of using a single binding region must be highly regulated to determine when and where β-catenin binds to different ligands. Evidence is accumulating that the N-terminal of β-catenin plays a key role in control β-catenin protein stability, subcellular localization and transcription activity [7].

Early studies show that β-catenin has two major pools: plasma membrane pool bound to E-cadherin which is relatively stable and cytoplasmic pool in a complex consisting of glycogen synthase kinase-3 (GSK-3), adenomatous polyposis coli (APC) and Axin that is dynamically balanced between degradation and accumulation. The degradation is ubquitin-mediated by sequential phosphorylation on S33, S37, T41 (by GSK3) and S45 (by CKI) in the absence of Wnt signaling. Recent work suggests that the N-terminal phosphorylation of β-catenin has functions beyond controlling protein degradation. β-catenin with phosphorylated S45 tends to accumulate in nucleus and spatially separates from isoform with S33/S37/T41 phosphorylation [8]. The S33/S37/T41/S45 phosphorylated β-catenin localizes at several subcellular sites, including cell-cell contacts where the phosphorylated β-catenin associates with E-cadherin at the adherens junction and with APC at cell protrusions [9], [10]. These data suggest that N-terminally phosphorylated β-catenin may serve distinct functions in nucleus and cell migration. A key consequence of the Wnt signaling is to generate and accumulate nuclear transcriptionally active β-catenin (ABC), which has been known as unphosphorylated at S37/T41 [11], [12]. However, this β-catenin isoform, recognized by a monoclonal antibody 8E7 [11], [12], is more readily detected at plasma membrane in complex with E-cadherin [13], [14]. In E-cadherin negative cells, this isoform is primarily monomeric in cytoplasm [14]. Upon Wnt stimulation, this isoform first moves to plasma membrane to form a complex with APC and LRP5 and then translocates to nucleus [13].

Protein Kinase D1 (PKD1), a serine/threonine kinase, has been implicated in numerous cellular functions, including cell survival, migration, differentiation, proliferation and cell-cell adhesion [15], [16]. PKD1 has been reported to be downregulated in advanced prostate, breast and gastric cancers and shown to play a role in tumorigenesis and metastasis [17], [18], [19]. Embryonic deletion of PKD1 in mice is lethal [20], suggesting PKD1 plays a crucial role in development, which cannot be replaced by other PKD family members, PKD2 and PKD3. We previously showed that PKD1 phosphorylates β-catenin at T112 and T120 [21]. The T120 residue is critical for binding to a-catenin since mutation of T120 to alanine abolished a-catenin binding [22]. Crystal structure analysis of β-catenin showed that the T120 residue locates at the beginning of a-catenin binding domain [5], [6]. Overexpression of PKD1 in prostate cancer C4-2 cell line, which has low endogenous PKD1 expression [23], suppresses epithelial to mesenchymal transition and tumor incidence in mice xenograft model [24]. PKD1 suppresses transcription factor Snail, a known E-cadherin repressor by inhibitory phosphorylation, induces expression of E-cadherin and up-regulates β-catenin [24]. However, high level of total β-catenin alone in C4-2/PKD1 cells is not sufficient to induce Wnt signaling [24]. Using a recently developed and characterized phosphor-threonine specific antibody that recognizes the phosphorylated T120 of β-catenin (pT120), we found that the nuclear β-catenin in C4-2/PKD1 cells is more reactive to the pT120 antibody and not recognized by the 8E7 antibody. In contrast, the nuclear β-catenin in C4-2 cells is not reactive to the pT120 antibody, but recognized by the 8E7 antibody. These results suggest that PKD1 activity influences the post-translational modifications and transcription activity of β-catenin via T120 phosphorylation. The pT120 antibody also reveals β-catenin accumulation in transGolgi network (TGN) in normal prostate tissue, but diminished in prostate cancer.

## Results

### PKD1 inhibits β-catenin transcription activity

**Figure 1:**
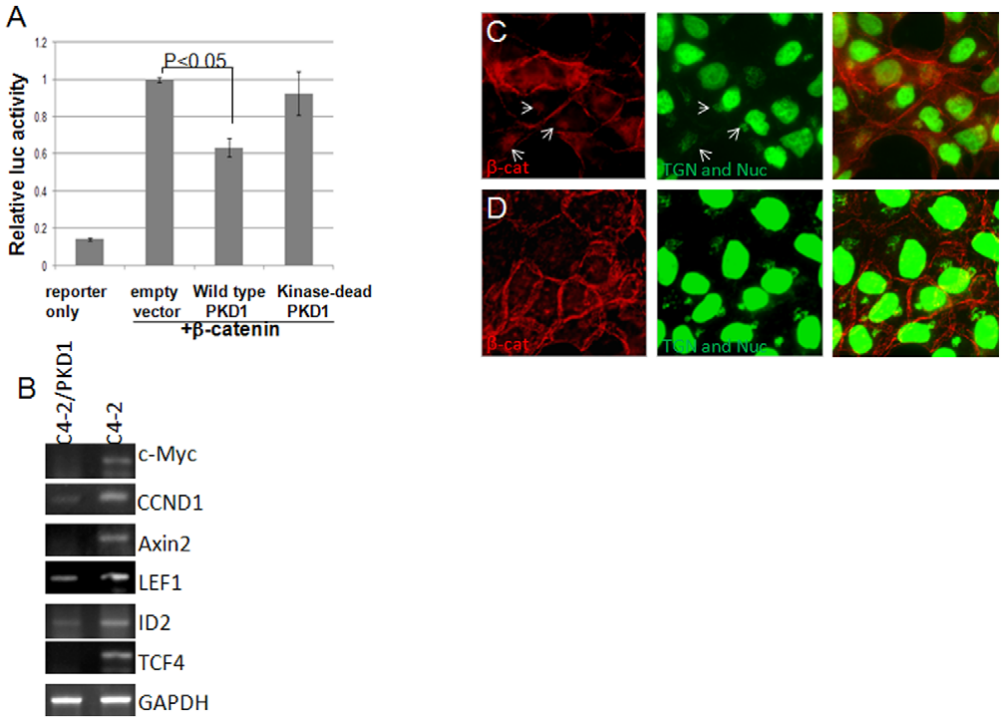
PKD1 represses Wnt/β-catenin transcription activity. (A) PKD1 inhibits β-catenin/TCF transcription activity. Topflash assay was performed in LNCaP cells. Data were normalized by *Renilla* luciferase activity based on average of triple samples. Error bars represent standard deviation. Significant difference between the control and tested groups were determined by Student's t-test. (B) Overexpression of PKD1 in C4-2 cells results in inhibition of Wnt target genes. Representative results of semi-quantitative RT-PCR for Wnt/β-catenin target genes from two independent assays (see Materials and Methods). (C) β-catenin in C4-2/PKD1 cells is co-localized with TGN marker p230 (white arrows). When the cells were treated with PKD1 small molecule inhibitor CID 755763 (50 nM) for 3days, the pattern is decreased (D). Anti β-catenin antibody H102 was used to stain endogenous β-catenin (red). TGN marker p230 and nuclei were labeled green. Images represent typical results from two tests.

We showed previously that overexpression of PKD1 suppressed cell proliferation and motility [21] and induced β-catenin membrane trafficking [28]. To study the influence of PKD1 on β-catenin function, we first measured β-catenin transcription activity using Topflash assay in the presence of PKD1. Wild type PKD1 was able to suppress the β-catenin/TCF transcription activity. In contrast, a kinase-dead mutant PKD1 (S744A/S748A) failed to inhibit the β-catenin/TCF transcription activity (Fig. 1A). Next, we compared expressions of endogenous Wnt target genes in prostate cancer C4-2 cell line and C4-2 cells that overexpress PKD1 (C4-2/PKD1). The high metastatic C4-2 cell line was derived from low metastatic LNCaP cell line by recovering tumor cells from metastatic sites in mice xenograft [29]. The C4-2 cell has lower endogenous PKD1 protein expression compared to parental LNCaP cells [17]. Semi-quantitative RT-PCR shows that Wnt/β-catenin target genes were expressed more in C4-2 cells than those in C4-2/PKD1 cells (Fig. 1B). These results suggest that Wnt/β-catenin is more active in C4-2 cells than in C4-2/PKD1 cells.

Since PKD1 directly phosphorylates β-catenin at threonine-112 and 120 residues, we examined the subcellular locations of β-catenin in the presence and absence of PKD1 activity. The endogenous β-catenin localizes to plasma membrane and a portion of cytoplasm β-catenin accumulates in transGolgi Network (TGN), where it co-localizes with TGN marker P230 (white arrows, Fig. 1C). Pre-incubation with a PKD1 small molecule inhibitor CID755673 [30], the accumulation of β-catenin in TGN becomes diminished (Fig. 1D).

### Development and characterization of pT120 antibody

Among the two PKD1 phosphorylation sites T112 and T120, T112 is also a site for casein kinase II (CKII) phosphorylation [31], PKD1 is the only known kinase to phosphorylate T120. Therefore, we chose the pT120 for developing phosphothreonine specific antibody (see Materials and Methods).

**Figure 2:**
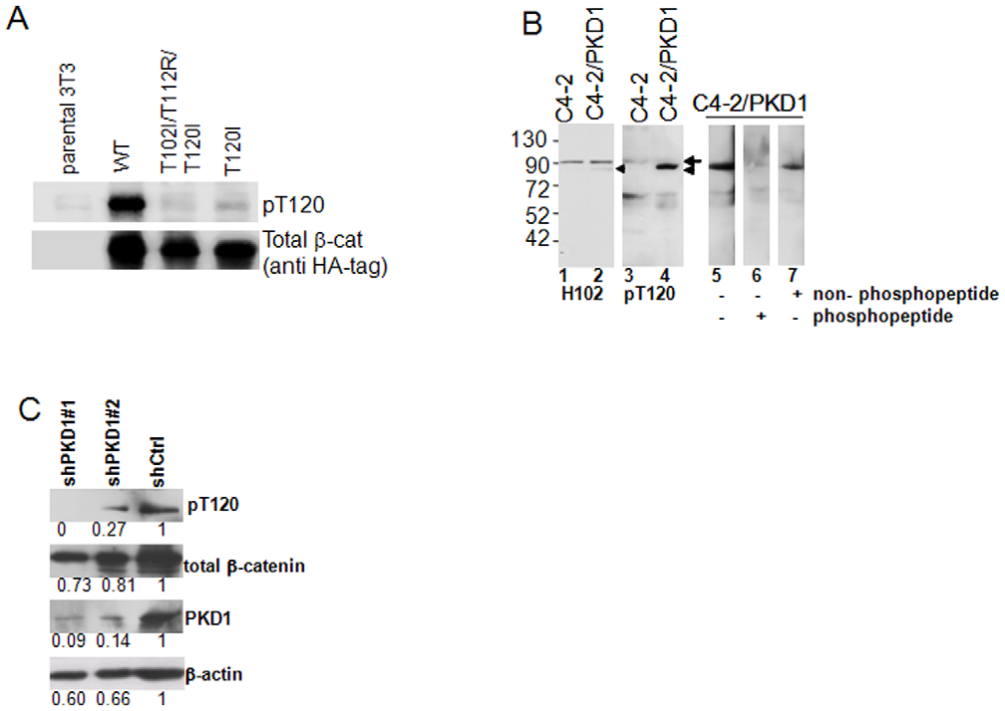
Characterization of pT120 antibody. (A) NIH 3T3 lysates that were transfected with either HA-tagged wild type, or T102I/T112R/T120I triple mutant, or T120I mutant β-catenin and blotted by pT120 antibody. (B) Cell lysates from C4-2 (lanes 1 and 3) and C4-2/PKD1 (lanes 2 and 4 to 7) cells were blotted with either β-catenin conventional antibody H102 (lanes 1 and 2) or pT120 antibody (lanes 3–7). Competition assay was performed in C4-2/PKD1 cell lysate in the presence of 20 nM of antigenic phospho-peptide (lane 6) or the nonphospho-peptide (lane 7). (C) Inhibition of PKD1 activity decreases T120 phosphorylation. LNCaP cells were transiently transfected with two shRNA targeting PKD1 or a control shRNA vector. Cell lysates were blotted with antibodies indicated. The relative protein levels quantified by densitometry are expressed as fraction of the control group on the average of two assays.

In order to confirm specificity of pT120 antibody, we first transfected HA-tagged wild type, T120I and T102I/T112R/T120I mutants of β-catenin into mouse 3T3 cell, a PKD1 positive cell line [32]. Cell lysates were used for Western blot. The pT120 antibody recognizes wild type β-catenin presumably phosphorylated, but not the mutants that lack T120 phosphorylation (Fig. 2A), suggesting the pT120 antibody specifically recognizes T120 phosphorylation.

Next, we compared the pT120 β-catenin in C4-2 and C4-2/PKD1 cells. Cell lysates from C4-2 cells and C4-2/PKD1 were blotted with either a conventional β-catenin H102 antibody (for detection total β-catenin) or pT120 antibody (Fig. 2B). The H102 antibody detects a major β-catenin band around 90 kDa in both C4-2 and C4-2/PKD1 cells and a minor band below 90 kDa in C4-2/PKD1 cell lysate only (arrowhead in lane 2). The pT120 antibody strongly reacts to the lower band in C4-2/PKD1 cell (arrowhead, lane 4) and weakly to the band at 90 kDa in C4-2 cells (arrow, lane 3). The results are consistent with our previous observation that the PKD1 phosphorylated β-catenin abnormally appeared at lower molecular weight, although phosphorylated proteins usually appear at higher molecular weights in most cases [21]. To further address the specificity of this pT120 antibody, we performed peptide competition assay. The antigenic phosphopeptide and non-phospho (normal) peptide were pre-mixed with the pT120 antibody individually and used to blot C4-2/PKD1 cell lysate. The phosphopeptide can compete with pT120 antibody binding (lane 6), but not the normal peptide (lane 7). Finally, knockdown PKD1 protein levels in LNCaP cells by shRNA resulted in lower pT120 antibody signal (Fig. 2C), suggesting that the T120 residue is a phosphorylation site for PKD1 in vitro. The data suggests that that pT120 antibody specifically recognizes the T120 phosphorylation of β-catenin.

### Overexpression of PKD1 blocks nuclear accumulation of active β-catenin

**Figure 3:**
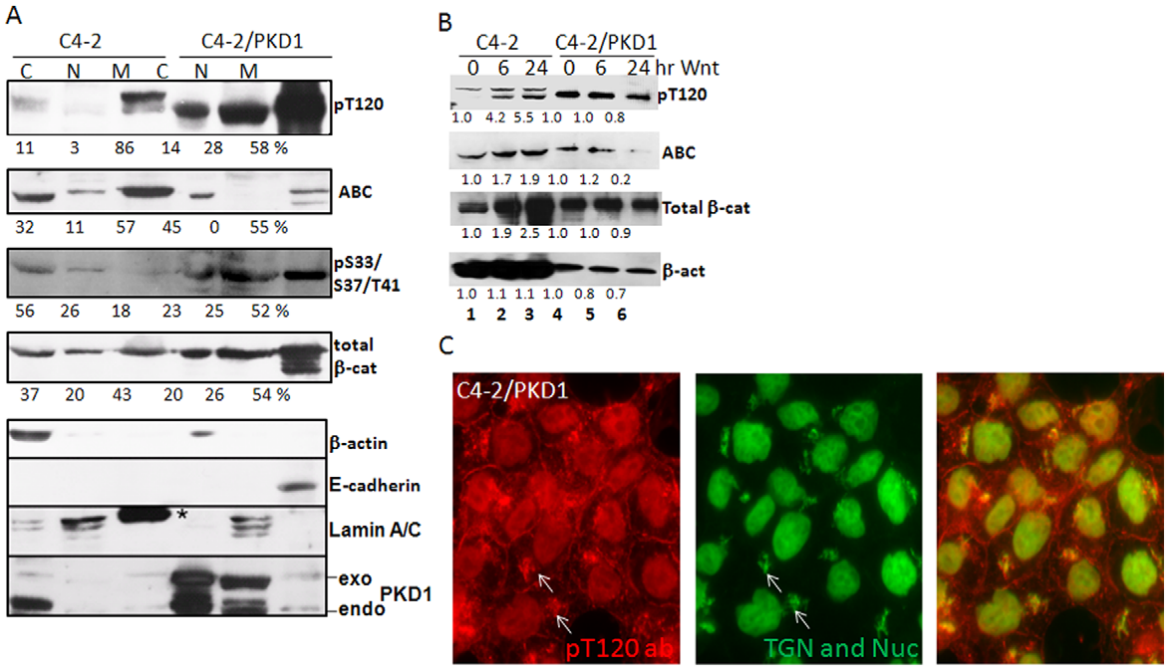
pT120 β-catenin blocks generation of nuclear ABC and accumulates in trans Golgi network. (A) C4-2 and C4-2/PKD1 cells were fractionated into fractions enriched for cytoplasmic, nuclear or membrane proteins and analyzed by Western blot with antibodies as noted. E-cadherin, lamin A/C and b-actin were used as plasma membrane, nuclear and cytosol markers, respectively. The * indicates a leftover band from previous blotting. The densitometric data of each β-catenin isoform in a cell was the average of three assays and was expressed as percentage. (B) Overexpression of PKD1 prevents Wnt-induced β-catenin accumulation. C4-2 and C4-2/PKD1 cells were cultured with recombinant Wnt3a for indicated times. The relative amount of β-catenin at each time point was expressed as a ratio to the amount of β-catenin at starting time 0 (data was from a single assay). (C) Immunofluorescence staining of C4-2/PKD1 cells with pT120 and TGN p230 antibodies The TGN and pT120 colocalization are indicated by white arrows.

To study the influence of PKD1 activity on β-catenin, we compared β-catenin subcellular distribution in C4-2 and C4-2/PKD1 cells. Subcellular fractions enriched with soluble cytosolic, nuclear or total membrane fractions (including organelle and plasma membranes) were prepared and blotted with pT120, 8E7 (for ABC), pS33/S37/T41, and H102 antibodies (Fig. 3A). In C4-2 cells, the pT120 β-catenin is predominately localized to total membrane fraction, with minor amounts in soluble cytosol and nucleus (lanes 1–3). In contrast, the pT120 β-catenin is readily detected in every subcellular fraction of C4-2/PKD1 cells (lanes 4–6). The active β-catenin detected by 8E7 antibody generally demonstrates an inverse relationship to pT120 β-catenin, i.e. high levels of ABC in C4-2, especially in nuclei (lane 2). The nuclear ABC is not detectable in the C4-2/PKD1 cells, although relative amount of nuclear β-catenin is comparable to C4-2 cells (20% vs. 26% in C4-2 and C4-2/PKD1 cells). Furthermore, phosphorylated S33/S37/T41 primarily present in C4-2/PKD1 correlates positively with pT120 distribution. Since the expression of Wnt target genes in C4-2/PKD1 cells is less than those in C4-2 cells (Fig. 1B), the nuclear β-catenin in C4-2/PKD1 cells, which consists of phosphorylated T120 β-catenin, is less transcriptionally active, suggesting that β-catenin protein level alone is insufficient to count signaling activity. Large differences in total β-catenin in cytoplasm and membrane fractions are also observed (Fig. 3A). Since the C4-2 is an E-cadherin negative cell and C4-2/PKD1 is an E-cadherin positive cell, it is reasoned that the β-catenin in C4-2/PKD1 cell is more membrane bound due to binding to E-cadherin and less likely to be found as soluble cytoplasm protein (compare Fig. 1C and Supplementary Fig. S1a). In addition, immunofluorescence image demonstrate that the pT120 β-catenin in C4-2/PKD1 distributes in cytosol, nucleus and plasma membrane and co-localizes with TGN marker p230 in cytosol (Fig. 3C), which is consistent with H102 staining pattern (Fig. 1C).

### Overexpression of PKD1 prevents Wnt-induced β-catenin accumulation

Although C4-2 cells do not express E-cadherin, C4-2/PKD1 is E-cadherin positive (Fig. 3A) due to inhibition of transcription factor Snail by PKD1 ([24]). Upon Wnt3a treatment, both total and active β-catenin increasingly accumulates in C4-2 cells after 6 and 24-hour stimulation (lanes 2 and 3, Fig. 3B). In contrast, total and pT120 β-catenin in C4-2/PKD1 cells remains unchanged before and after Wnt stimulation (lanes 4–6), active β-catenin remains nearly unchanged after 6 hours treatment, prolonged treatment (24 hours) actually decreases the amount of ABC (lane 6). Hendriksen et al. reported similar findings in mammary epithelial cell cultures and suggested that the E-cadherin/β-catenin membranous complex resists Wnt stimulation [13]. Interestingly, the known patterns of pT120 in un-stimulated C4-2 and C4-2/PKD1 cells, i.e., a high molecular weight band in C4-2 and a low molecular weight band in C4-2 PKD1 (lanes 3 and 4, Fig. 2B) remain relatively unchanged throughout Wnt treatment. However, a new band recognized by pT120 antibody appears and accumulates in C4-2 cells after Wnt treatment up to 24 hours (lanes 2 and 3).

### The pT120 β-catenin accumulates at TGN of prostate epithelial cells

We previously observed that PKD1 activity is associated with β-catenin membrane trafficking and the two proteins co-localize with multiple TGN markers [28], suggesting that pT120 β-catenin may involve in β-catenin membrane trafficking. Immunofluorescence staining with pT120 antibody in various prostate epithelial cells reveals that pT120 subcellular localization has different patterns from H102 antibody. In C4-2 cells, which are E-cadherin negative, total β-catenin level is low and cytosol (Supplementary Figure S1a), pT120 β-catenin mainly localizes to paranuclear area. In E-cadherin positive cells, including C4-2/PKD1, LNCaP, RWPE1 and BPH-1 cells, the H102 antibody staining reveals β-catenin at plasma membrane (Fig. 1C and Supplementary Figure S1b–d). In addition, the pT120 antibody shows different localization patterns: , In C4-2/PKD1 cells, the pT120 β-catenin distributes to cytosol, nucleus and plasma membrane and the cytosol pT120 β-catenin co-localizes with TGN (Fig. 3C). In LNCaP and RWPE1 cells, the pT120 β-catenin distributes predominately as diffused cytosol protein (Supplementary Fig. S1b and S1c). However, in a benign hyperplastic prostate epithelial BPH-1 cell line, the pT120 β-catenin shows predominate staining on plasma membrane and nucleus (Supplementary Figure S1d). The results suggest that the variation of subcellular localization of pT120 may be sensitive to cellular signaling changes in prostate cancer progression.

### T120 phosphorylated β-catenin accumulates in TGN in prostate tissue

**Figure 4:**
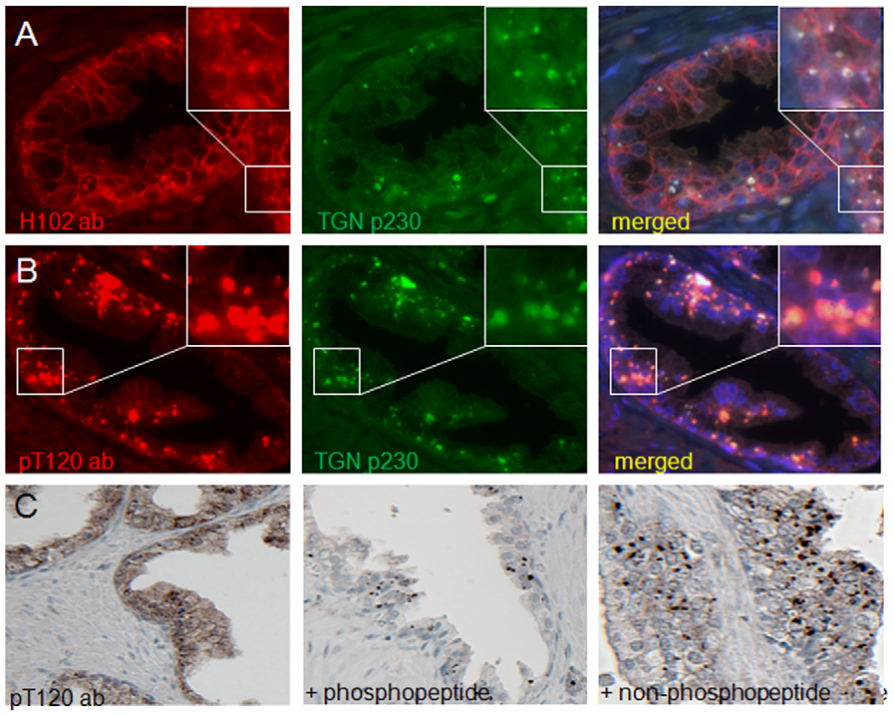
pT120 β-catenin localizes in TGN in normal prostate tissue. Formalin fixed and paraffinized normal human prostate tissues were stained with either β-catenin H102 (A) or pT120 (B) antibodies with TGN marker p230 antibody. Nucleus was stained in blue. Inserts are higher magnification for details. Representative images from three assays were shown. (C) Immunohistochemical staining with the pT120 antibody on normal prostate tissue (left), in the presence of 20 nM antigenic phospho-peptide (middle) or 20 nM nonphospho-peptide (right).

To study the pT120 β-catenin *in situ*, we co-stained formaldehyde-fixed, paraffin embedded benign human prostate tissue using the pT120 or H102 antibody with TGN marker p230. β-catenin visualized by conventional H102 antibody typically localizes on plasma membrane as well as TGN (Fig. 4A). In contrast, vast majority of β-catenin visualized by the pT120 antibody co-localizes with p230 on TGN (Fig. 4B). Weak staining of plasma membrane is also detected.

To confirm the specificity of pT120 antibody using in immunohistochemetry (IHC), we performed peptide competition assay on pT120 antibody in IHC. The pT120 antibody staining displays a more diffuse pattern with heavy particles in cytoplasm presumably the TGN (left panel, Fig. 4C). In the presence of antigenic phosphopeptide, most staining disappeared except few spots in TGN (middle panel, Fig. 4C), the most likely explanation for the observation is that the TGN local concentration of pT120 β-catenin is too high for the phosphopeptide to totally compete. In contrast, the non-phosphopeptide cannot compete with pT120 antibody staining in cytoplasm but not in TGN (right panel, Fig. 4C).

### The T120 phosphorylated β-catenin in TGN dramatically decreased in prostate cancer specimens

**Figure 5:**
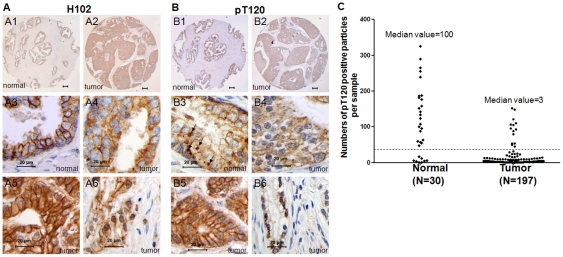
pT120 β-catenin is significantly decreased in TGN of prostate cancer samples. Immunohistological staining was performed with H102 (panel A) and pT120 (panel B) antibodies on serial section of tissues. 3 and 4 are higher magnification of 1 and 2, respectively. 1 and 3 are normal prostate tissue. 5 and 6 show that pT120 β-catenin predominantly distributes to plasma membrane and nucleus in tumor samples. Bars in panels 1 and 2 equal to 50 micrometer. (C) Scatter plot for pT120 staining pattern in normal and prostate cancer samples. Normal and tumor samples were counted for the “particle” staining pattern as described in Fig. 4B and 5B3. The number on Y-axis represents the average of two independent counts. A cutoff at 30 (dashline) is used to as arbitrary definition of positive and negative staining.

In order to explore utility of T120 β-catenin antibody in clinical specimens immunohistochemical (IHC) studies were carried out. IHC was performed on serial sections of paraffinized human prostate normal and cancer samples arranged on tissue arrays to compare pT120 β-catenin to total β-catenin distribution (Fig. 5A1, A2, B1 and B2). In normal samples, the pT120 antibody typically showed diffuse cytoplasm staining with heavy TGN particles (arrows, Fig. 5B3). Twenty-two of 30 (73.3%) benign prostate tissues demonstrated pT120 β-catenin in TGN (Table 1) as judged by over 30 TGN particles. In contrast, 183/197 (92.9%) cancer specimens showed no pT120 β-catenin accumulation in TGN compared to benign prostate tissues (Fig. 5C, p<0.001). Cancer specimens with Gleason grade ≥7 showed even lower percentage of positive TGN staining (6.3%) versus 14.8% positive staining from specimens with Gleason 3–6. Table 1 summarizes the finding of pT120 antibody staining in human prostate cancer tissues. In addition, two samples contain both tumor tissues and adjacent normal glands. The pT120 antibody staining shows that pT120 β-catenin only accumulates in tumor adjacent normal glands (arrows, Supplementary Figure S2), not in tumor tissue.

**Table 1:** Analysis of pT120 antibody staining in prostate cancer.

	TransGolgi network staining			Two-sample *t* test (*P* value)	
	n	positive (%)	negative (%)	Normal vs. tumor	Gleason 3–6 vs. 7–10
Normal	30	22 (73.3%)	8 (26.7%)		
Tumor	197	14 (7.1%)	183 (92.9%)	P<0.001	
Gleason					
3 to 6	27	4 (14.8%)	23 (85.2%)		
7 to 10	170	10 (5.9%)	160 (94.1%)		P<0.05

Other abnormal pT120 antibody staining patterns also found in prostate cancer samples, including strong plasma membrane (Fig. 5B4) and nuclear staining (Fig. 5B5), which may resemble patterns found in BPH1 cells (Supplementary Figure S1c). Table 2 summarizes the finding of these abnormalities.

**Table 2:** Comparison of H102 and pT120 staining patterns.

	Total	H102 staining	pT120 staining
**Normal tissue**			
membranous ββ-catenin	9	8 (88.9%)	
TGN β-catenin	9		8 (88.9%)
**Tumor tissue**			
increased expression	56	18 (32.1%)	9 (16.1%)
decreased expression	56	5 (8.9%)	5 (8.9%)
membranous	54	42 (77.8%)	8 (14.3%)
cytoplasm/nuclear	54	12 (22.2%)	6 (10.7%)

## Discussion

PKD1 suppresses cancer cell epithelial to mesenchymal transition by inhibitory phosphorylation of transcription factor Snail, a known E-cadherin repressor. Overexpression of PKD1 in C4-2 induces expression of E-cadherin and lowers tumor incidence in mice xenograft models [24]. β-catenin serves as a co-repressor of expression of prostate cancer metastasis repressor gene *KAI1 (CD82)* [33]. We have previously shown that expression of *KAI1* actually was induced in C4-2/PKD1 cells, although the expression of β-catenin is also upregulated [24]. These data suggest that β-catenin protein levels do not necessarily correlate with its transcription activity. In this study, we found that the protein level of nuclear β-catenin in C-42/PKD1 is more compared to C4-2 cells (Fig. 3B), but β-catenin transcription is lower in C4-2/PKD1 cells (Fig. 1B). This finding cannot be merely explained by induction of E-cadherin expression, because E-cadherin is thought to sequester cytoplasm β-catenin pool and decrease availability of free β-catenin for transcription in nucleus. The pT120 antibody unveils the biochemical identity of nuclear β-catenin in C4-2/PKD1 is T120 phosphorylated (also S37/T41 phosphorylated). In contrast, nuclear β-catenin in C4-2 is S37/T41/T120 unphosphorylated. These findings suggest a possible correlation among phosphorylation of these sites and crosstalk between PKD1 and GSK3 signaling. A novel β-catenin pool in TGN is also unveiled and is consistent with our previous finding that PKD1 and β-catenin co-localized at TGN by using five TGN and transport vehicle markers [28].

Although the critical role of Wnt signaling is demonstrated in the mutations of APC or β-catenin gene, such mutations are infrequent in prostate cancer, which counts 16% and 5%, respectively [34]. Mutations of Axin1 in prostate cancer are also reported [35]. In contrast, many studies have examined the alterations in β-catenin expression and localization as a potential new prostate cancer biomarker and the results vary from study to study (see Table I in Ref. [35]). β-catenin immunostaining using a conventional β-catenin antibody reveals that about 20–71% in prostate cancer specimen have abnormal expression patterns of β-catenin [36], [37]. We observed 32.1% (18/56) samples have increased β-catenin expression using H102 antibody (Table 2). In contrast, we observe that 93% prostate cancer samples have decreased pT120 β-catenin accumulation in TGN in both low and high Gleason grade (Table 1). If this pT120 staining pattern truly reflects PKD1 activity *in vivo*, then the results would be interpreted that 93% prostate cancer samples have less PKD1 activity. Interestingly, reduced expression of PKD1 was found in more than 95% of human invasive breast tumors examined by antibody staining [19]. In 59% gastric cancer samples examined, PKD1 has a >2-fold decrease in expression due to epigenetic silencing.

Our work reveals a new regulatory step of the dynamic equilibrium of free and bound β-catenin. The phosphorylation of T120 correlated inversely with unphosphorylated S37/T41 ABC. The distribution of pT120 β-catenin is distinct in normal and malignant prostate tissues. With malignant transformation, staining of pT120 at TGN is dramatically decreased. We propose that β-catenin T120 phosphospecific antibody can be used to study alteration in subcellular distribution of β-catenin and Wnt signaling, and possibly as biomarker in prostate and other tissues, which may facilitate risk stratification or predict disease outcomes. Further investigations on the phosphorylation relationship of T120 and S37/T41 may help to elucidate the role PKD1 in the regulation of Wnt/β-catenin signaling.

## Materials and Methods

### Development of the pT120 antibody

An antigen peptide correspondent to the β-catenin T120 region, _109-_IPSTQFDAAHPpTNVQRLAEPSQML (where pT is phosphothreonine-120) was synthesized. The peptide was injected into two rabbits to raise antisera. A corresponding nonphospho-peptide was also synthesized for antibody screening and purification. The peptide synthesis and antibody generation was performed by commercial contractor Millipore (Billerica, MA).

### Western blotting and protein sample preparation

To prepare whole cell lysates, cells cultured on monolayer were washed by phosphoate buffered saline (PBS) and dissolved in RIPA buffer [25] (50 mM Tris-HCl, pH7.4, 150 mM NaCl, 0.5% sodium deoxycholate, 0.1% SDS and 1% NP-40) plus protease inhibitor cocktail (Sigma Chemicals, cat# P-8340). For fractionation experiments, a commercial cell fractionation kit (the ProFEK kit from ITSI Biosciences) was used according to manufacturer's recommendation. Briefly, cultured cells (5×10^6^) were collected by scratching and washed with PBS plus a protease inhibitor cocktail and a phosphatase inhibitor cocktail (A.G. Scientific, cat #P-1517). Cell pellet was resuspended in 250 microliter of a hypotonic buffer (Cytosol Buffer 1) and homogenized with a plastic pestle. After incubation 15 min on ice, cells were centrifuged at 8,000×g for 5 min. The supernatant was collected and centrifuged again at 16,000×g for 10 min and supernatant is used as soluble cytosol fraction. The initial pellet was washed with a wash buffer (Wash Buffer 2), spinned down, resuspended in 125 microliter of Nuclear Buffer 3 and homogenized. After incubating on ice for 30 min, the lysate was centrifuged at 16,000×g for 10 min. The supernatant was collected as nuclear-protein enriched fraction. The resulting pellet was resuspended in 250 microliter of Total Membrane Buffer, homogenized and incubated on ice for 30 min. The lysate was centrifuged at 16,000×g for 10 min and the supernatant was collected as membrane-protein enriched fraction. It is worth noting that the total membrane fraction generated by the kit includes plasma membrane and cellular membrane. The protein samples were separated on 10% SDS-PAGE and transferred to a polyvinylidene difluoride membranes (PVDF) using a semi-dry transfer devise (Thermo Scientific) at constant current model (0.2 A). The membrane was then incubated with TBST (50 mM Tris-HCl, pH7.4, 100 mM NaCl and 0.2% Tween-20) plus 3% nonfat dry milk to block non-specific binding for 1 hour at room temperature. Incubation with primary antibodies (see Reagents section) was performed at 4 C for overnight. The densitometric quantification of immunoblotting was carried out with Image J software.

### Reagents

For western blotting, the pT120 antiserum was used at dilution of 1∶2000. For peptide competition assay, the phospho- or nonphospho-peptides were added in the pT120 antibody solution to a final concentration of 20 nM prior to blotting. A commercial β-catenin antibody (H102, Santa Cruz Biotechnology) was used for comparison at 1∶2000 dilution. The phospho-S33/S37/T41 β-catenin antibody (Cell Signaling,) was used at dilution of 1∶200 for western blot. Monoclonal antibody 8E7 (Millipore) that recognizes unphosphorylated S37/T41 β-catenin was used at 1∶800 for blotting. The HA-tagged wild type β-catenin in pCS2+ mammalian expression vector, T120I and T102I/T112R/T120I mutants were described previously [21]. The shRNA constructs targeting PKD1 were purchased from Open Biosystems/Thermo Scientific (Waltham, MA). Human recombinant Wnt-3a was purchased from R&D Systems and used at 50 ng/ml in cell culture [26].

### Cell cultures, treatment and Topflash assay

Prostate cancer cell lines LNCaP (clone FGC, from ATCC) C4-2 (Urocor) and a benign prostate hyperplastic epithelial cell line BPH-1 (kindly provided by Dr. Lucia Languino, Thomas Jefferson University) were cultured in RPMI1640 medium plus 10% bovine fetal serum (Invitrogen). C4-2 cells that stably express PKD1-GFP (C4-2/PKD1) was previously described [27]. Normal prostate epithelial cell line RWPE-1 (ATCC) was maintained in Keratinocyte Serum Free Medium (K-SFM, Invitrogen) and supplied with bovine pituitary extract (Invitrogen) and human recombinant epidermal growth factor (EGF, 5 ng/ml. Invitrogen). Mouse NIH 3T3 cell line was cultured in DMEM medium with 10% FBS. Transient transfection was performed by Lipofectamine 2000 reagent (Invitrogen) overnight. Topflash assay conditions were described previously [21]. Briefly, LNCaP cells (2×10^4^ cell/well in a 96-well plate) were transfected by 50 ng Topflash reporter, 5 ng Renilla luciferase vector and 100 ng either PKD1 expression vector or empty vector control for overnight. Luciferase activity was measured 2 days later. Relative luciferase activity (RLU) was obtained by averaging of normalized firefly luciferase activities from three samples. Normalization was performed with Renilla luciferase from Promega (Madison, WI).

### Immunofluorescence and immunohistochemistry

For immunofluorescence, cultured cells were fixed in 5% formalin in PBS for 10 minutes at room temperature. Human normal prostate tissues were deparafinized and rehydrated. The pT120 and H102 antibodies were diluted 2000-fold in PBS with 0.1% Triton X-100 and 10 mg/ml BSA. Antibody to transGolgi Network marker P230 (BD Transduction Laboratories) was used at a dilution of 1∶500. FITC and Cy3 labeled second antibodies (Jackson ImmunoResearch, West Grove, PA) were used at dilution of 1∶1,000. For immunohistochemistry, the pT120 and H102 antibodies were used at 1∶1000 and 1∶100 dilutions, respectively. Human prostate tissue arrays were purchased from US Biomax. Fluorescence and IHC images were taken in an Olympus IX-51 microscope and a Nickon Eclipse 50i microscope equipped with SPOT software, respectively. The assessment of β-catenin expression in tissue specimens was done by comparison of the average staining intensity of normal samples to the staining intensities of tumor samples, and described higher or lower intensity as up regulation or down regulation respectively. To score pT120 staining in trans Golgi Network, we counted 30 normal prostate tissues for the “particle” staining pattern as described in Figure 4 and Figure 5B3 by two reviewers independently and average number of stained particles in the whole core of each tissue was counted. Tumor tissues were defined as positive or negative by an arbitrary cutoff of 30 particles per core.

### Semi-quantitative RT-PCR

Total RNA was extracted with Trizol reagent (Invitrogen). Forty micrograms total RNA were used for reverse transcription in a 40 µl volume using SuperScript II kit ((Invitrogen) at 45 C for 1 hour. After RT, the reaction was diluted with TE buffer (Tris-HCl, 10 mM, pH8.0, EDTA 1 mM) to 200 microliter. Two microliter of the RT product was used for each PCR reaction. Thermocycle setting is 94 C for 2 minutes, 94 C for 30 seconds, 55 C for 45 seconds and 72 C for 30 seconds. PCR samples were taken at 27, 29, 31 and 33 cycles and analyzed by agarose gel electrophoresis. Wnt target genes were selected from a list in the Wnt Homepage at http://www.stanford.edu/%7ernusse/pathways/targets.html. Primers used are, *c-Myc*, For: ACCAGCTGGAGATGGTGACCGAG; Rev: CCGAGGACGGAGAGAAGGCGCT

*cyclin D1*, For: TGCTGAAGGCGGAGGAGACCTGC; Rev: CAGCAGCTCCTCGGGCCGGATGG

*Axin2*, For: TTATGCTTTGCACTACGTCCCTCCA; Rev: CGCAACATGGTCAACCCTCAGAC

*LEF1*, For: TTCAAGGACGAGGGCGATCCTCAAG; Rev: CGAGTTATTCGGGTACATAATGA

*ID2*, For: AACAGCCTGTCGGACCACAAGC; Rev: GGCTGACAATAGTGGGATGCG

*TCF1*, For: TGGACAAGGGGGAGTCCTGC; Rev: GGTTGAGGCCAGTGGTATCG

*GAPDH*, For:AGAAGGCTGGGGCTCATTTG; Rev: AGGGGCCATCCACAGTCCTTC

## Supporting Information

Figure S1 **Total and pT120 β-catenin localizations in cultured prostate cell lines.** (a) C4-2 cells, (b) RWPE1 cells, (c) LNCaP cells and (d) BPH-1 cells.
(TIF)

Figure S2 **pT120 β-catenin staining in prostate tumor and adjacent normal area.** A tissue core contains both tumor and normal tissues. The pT120 antibody staining reveals that pT120 β-catenin accumulates in TGN in the normal tissues (arrows, lower left). In adjacent tumor tissue, the pT120 β-catenin is more diffused in cytoplasm (upper left).
(TIF)
