# Supplementary figures and images for: Beta-Catenin Phosphorylated at Threonine 120 Antagonizes Generation of Active Beta-Catenin by Spatial Localization in trans-Golgi Network
Source: PLoS One. 2012 Apr 12;7(4):e33830. doi: 10.1371/journal.pone.0033830 (PMC3325232; doi:10.1371/journal.pone.0033830)

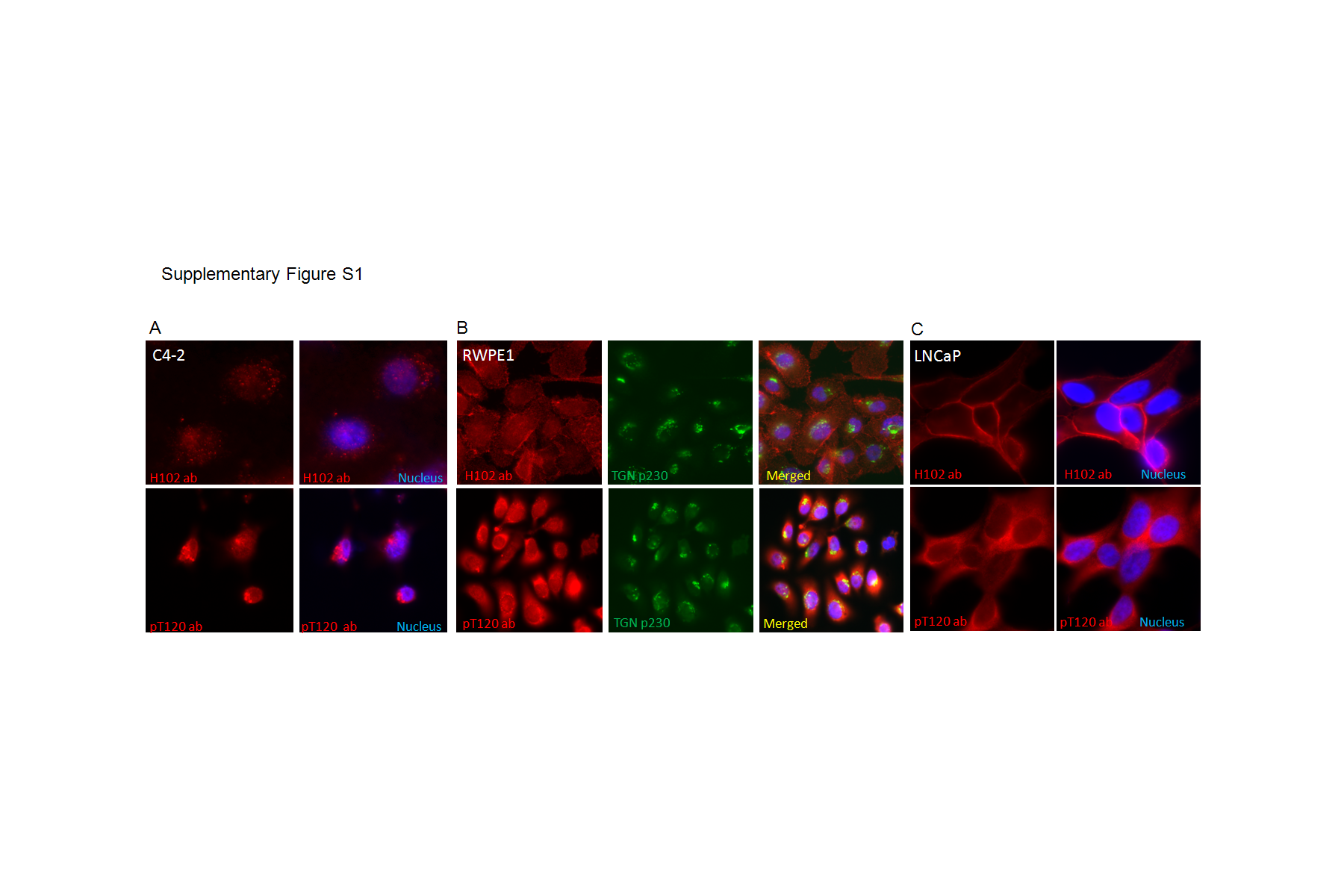

Supplement: Figure S1 — Total and pT120 β-catenin localizations in cultured prostate cell lines. (a) C4-2 cells, (b) RWPE1 cells, (c) LNCaP cells and (d) BPH-1 cells. (TIF) [file pone.0033830.s001.tif]

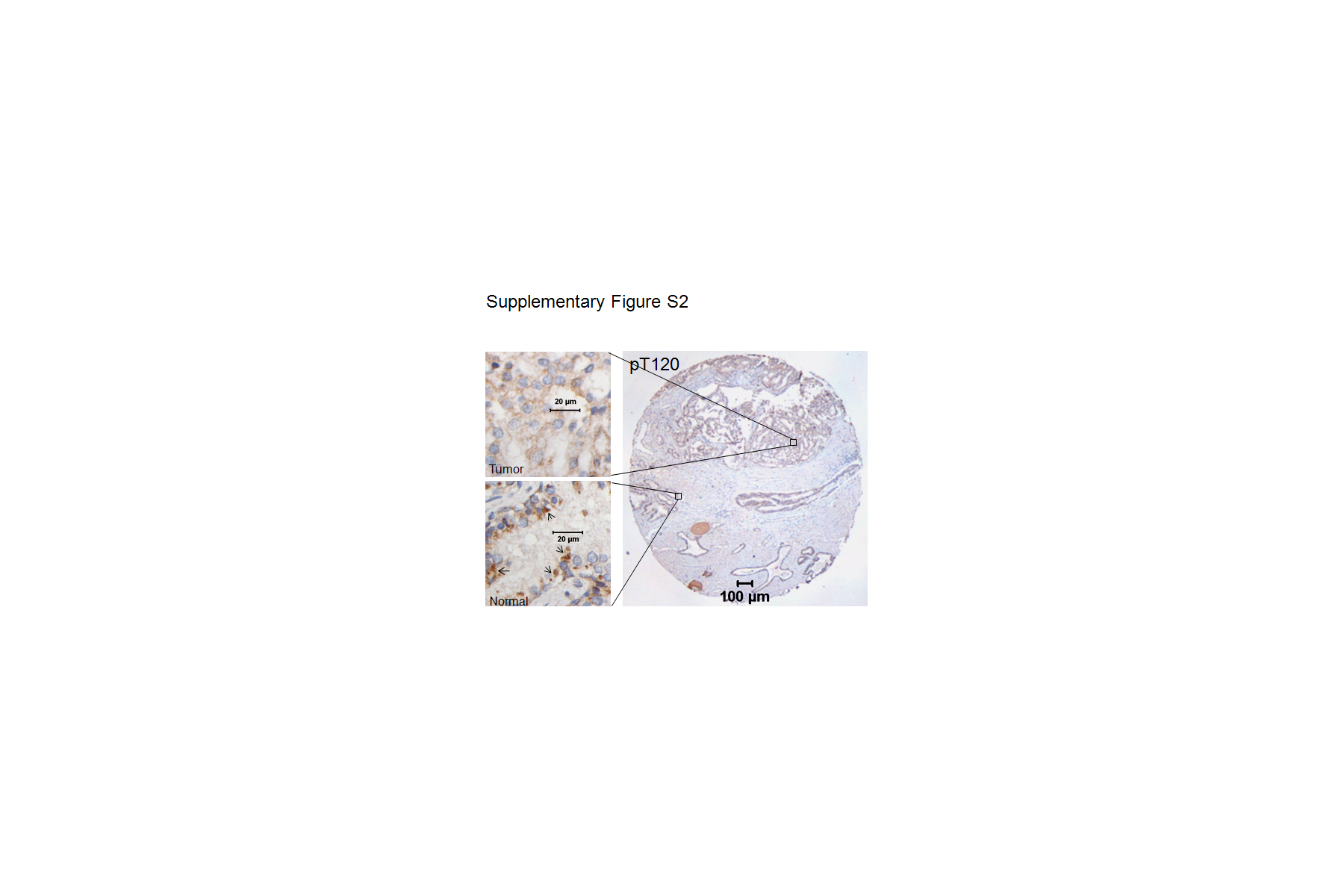

Supplement: Figure S2 — pT120 β-catenin staining in prostate tumor and adjacent normal area. A tissue core contains both tumor and normal tissues. The pT120 antibody staining reveals that pT120 β-catenin accumulates in TGN in the normal tissues (arrows, lower left). In adjacent tumor tissue, the pT120 β-catenin is more diffused in cytoplasm (upper left). (TIF) [file pone.0033830.s002.tif]
